# Laser Supported Reduction of Specific Microorganisms in the Periodontal Pocket with the Aid of an Er,Cr:YSGG Laser: A Pilot Study

**DOI:** 10.1155/2015/450258

**Published:** 2015-03-23

**Authors:** N. Gutknecht, C. Van Betteray, S. Ozturan, L. Vanweersch, R. Franzen

**Affiliations:** ^1^Department of Conservative Dentistry, Periodontology, and Preventive Dentistry, RWTH Aachen University Hospital, Pauwelsstraße 30, 52074 Aachen, Germany; ^2^Private Practice Konrad-Adenauer-Platz 8, 40764 Langenfeld, Germany; ^3^Department of Periodontology, Faculty of Dentistry, Biruni University, Topkapı, 34010 Istanbul, Turkey

## Abstract

*Objective*. The aim of this study was to evaluate the effectiveness of a radial firing tip of an Er,Cr:YSGG laser as an adjunct to a nonsurgical periodontal treatment.* Methods*. Twelve patients with chronic or aggressive periodontitis were treated by conventional periodontal treatment using ultrasonic devices and hand instruments and, additionally, in two quadrants with an Er,Cr:YSGG laser. A new radial firing tip (RFPT 14-5, Biolase) was used with 1.5 W, 30 Hz, 11% air, 20% water, and pulse duration 140 *μ*s. Microbiological smears were taken before treatment, one day after lasing, and three and six months after lasing. Pocket depths of all periodontal sites were measured before and six months after treatment.* Results*. The total bacterial load of* Prevotella intermedia*,* Tannerella forsythia*,* Treponema denticola*,* Fusobacterium nucleatum*,* Porphyromonas gingivalis*, and *Aggregatibacter actinomycetemcomitans *inside the pocket was reduced significantly throughout the whole examination time. Greater pocket depth reductions were observed in all groups. There was a slight higher reduction of pocket depth in the lased group after six months.* Conclusions*. These results support the thesis that Er,Cr:YSGG laser supported periodontal treatment leads to a significant reduction of periopathogenes and thereby helps the maintenance of periodontal health.

## 1. Introduction

The therapy of periodontal diseases has become one of the major fields in modern dentistry. 52.7% of the German population are infected with this disease and even 39.8% suffer from an aggressive kind of periodontitis. The British population is even more affected: 62% of the population are infected with moderate periodontal disease [1].

Gilthorpe et al. [2] described a sequential deterioration and repair that occur at the individual tooth sites over time as disease progression function. This phenomenon is individually accelerated or decelerated by different factors. One can differentiate between risk factors that are modifiable by the individual and/or the dentist and the ones that are nonmodifiable [3].

Nonmodifiable factors influence naturally the onset of periodontitis as well as the progression. They are

- age/gender/ethnicity,
- genetical preconditions (immune system, saliva),
- systemical diseases (diabetes mellitus, HIV, osteoporosis, and leukaemia). For diabetes mellitus, there is evidence that both Type-I and Type-2 diabetes have higher prevalence, extent, and severity of periodontal disease [4].

Modifiable factors are as follows.

*(i) Socioeconomic Indicators*. Evidence suggests that education has a greater influence than income in favourably affecting the level of periodontitis in the population [5].

*(ii) Specific Microbiota*. The consensus report of the 1996 World Workshop in Periodontics identified three species* Aggregatibacter actinomycetemcomitans*,* Porphyromonas gingivalis*, and* Tannerella forsythia* as causative factors of periodontitis [3]. The authors also suggest that these three species cannot be considered the only causative pathogens but are rather the ones for which sufficient data have accumulated. In common knowledge,* Prevotella intermedia, Treponema denticola,* and* Fusobacterium nucleatum* ssp. are also mentioned as leading germs for periodontitis.

*(iii) Cigarette Smoking* is one of the key factors for the deterioration of the healing process, which means in the same time that the disease progression is promoted by smoking habits [6].

The only factor, which can be influenced by the dentist, besides enlightenment, is the bacterial population inside the periodontal pockets. Since this is one of the defined major risks, the dentists' work is a key factor in diagnosis, therapy, and maintenance of the periodontal condition of the patient.

This study concentrates on two types of periodontitis corresponding to the classification of periodontal diseases according to the 1999 international workshop for classification of periodontal diseases and conditions in Oak Brook (Illinois, USA): Type II: chronic periodontitis, Section B: generalized type and Type III: aggressive periodontitis, Section B: generalized type.

Just like Socransky et al. [7], Borrell and Papapanou [3] pointed out the key factor in periodontal treatment besides enlightenment of the patient; the reduction of the pathogen microorganisms is the overall aim of periodontal treatment for the dentist. These microorganisms are the reason for inflammatory chain reactions in the gingiva, which lead to the loss of gingival attachment to the tooth and in the end to the loss of the tooth.

In science, it has been proven that laser treatment has a bactericidal effect on dental tissue. Different wavelengths have been analyzed. The newest investigations since the beginning of this century show promising results concerning the Erbium, chromium doped Yttrium-Scandium-Gallium-Garnet laser (hereinafter Er,Cr:YSGG laser). This present study evaluates the capability of the Er,Cr:YSGG laser with a wavelength of 2,780 nm and a 360° firing elastic tip to be the appropriate tool to reduce pathogenous microorganisms in the oral cavity and to eliminate the biofilm on the diseased root surfaces and the infected gingiva around the tooth in addition to a nonsurgical conservative periodontal treatment by being comfortable to work with for the dentist and being nearly pain free for the patient. This special tip is able to work on the hard tissue side inside the periodontal pocket on the one hand, which is to scale the root surface as well as to treat the soft tissue side of the pocket: (1) deepithelize the gingiva from the junctional epithelium, (2) sterilize the pocket exudates and the connective tissue, and (3) carbonize the opened blood vessels.

## 2. Materials and Methods 

### 2.1. Selection of Patients

In this study, 12 patients with a chronic or aggressive periodontitis have been examined and treated following the same treatment plan. In each quadrant, one could find pocket depth of at least 4–6 mm.

4 patients were females; 8 were males. Smoking was not an excluding factor for this study design. All patients had no further systemic diseases or medicamental treatment. They did not have periodontal treatment within the last 3 years and also had not taken antibiotics for the last 3 months before the start of the examination and throughout the study time. The patients were asked not to use any chlorhexidine within the treatment period.

### 2.2. Therapy Protocol

One week before measuring the pocket depths and taking the microbiological probe, the patients were in the office to see the dental hygienist for a professional supragingival dental cleaning. In this session, the oral hygiene index and the sulcus bleeding index were evaluated. The second appointment was the periodontal charting and the microbiology.

Two weeks after this pretreatment, all patients had a nonsurgical subgingival scaling with a sonic scaler (Kavo Sonicflex 2003 L, Biberach, Germany) and gracey curettes (Hu-friedy, Chicago, Illinois, USA) for all teeth. This scaling and root planning happened in a 24-hour time frame for optimal conditions. In addition, two quadrants were treated with a Waterlase MD Er,Cr:YSGG laser (Biolase, Irvine, California, USA). Those two quadrants were randomly distributed within the upper and lower jaw, right or left side, similar to a split mouth design, so that either the first and fourth quadrant or the second and third quadrant would get a laser treatment additionally.

The irradiation was repeated three times, each in a seven-day period for the same two quadrants. The pockets were lased in the bottom-up technique, which means the tip was placed at the bottom of the pocket and moved slowly coronal by circulating parallel to the surface of the teeth. The first postoperation microbiological smear was taken one day after the third lasing. This smear is taken only from the two quadrants which had the laser treatment.

Three months later, the patients have seen the dentist again for a microbiological smear, a hygiene session, and the oral indices. The last recall within this study was six months after treatment. In this session, another microbiological smear was taken and the pocket depth was determined again. The oral indices, that were reviewed each time the patient came to the office, were only taken to control the oral hygiene at home to exclude an error, which would be the lack of adequate oral hygiene.

### 2.3. Laser Settings

**Figure 1:**
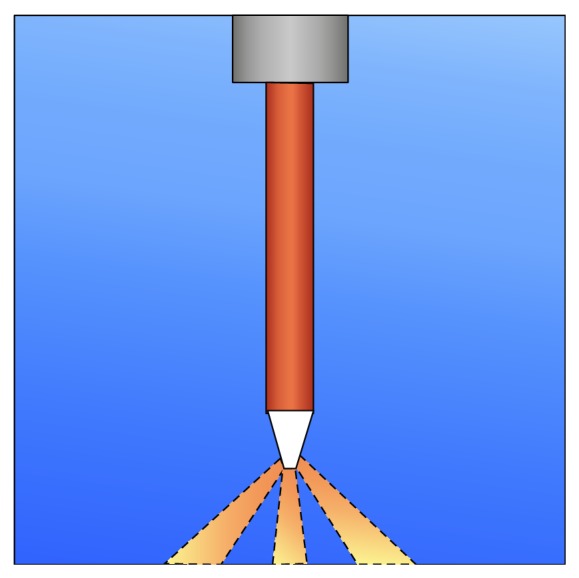


The MD standard handpiece was used with the new RFPT 5-14 tip. This tip has a new and special shape. It allows firing 360° to an irradiation cone as well as straight at the same time. It has a diameter of 580 *μ*m and a length of 14 mm. It produces primarily radial emission (80%) of laser energy with a portion of straight emission (20%) as seen in Figure 1 [8]. The company also accentuates the better access to the narrow part of the periodontal pocket since the tip has no side edges and is rather flexible. Another advantage of this radiation pattern is the significantly reduced power directly in front of the tip end. That may reduce a potential risk of damaging periodontal ligament within the pocket. At the same time, the efficiency of laser power emitted radially away from the end of the tip is increased for more efficient radiation of the root surface and soft tissue of the gum.

The very end of the tip has a diameter of 100 *μ*m. The outcoming radial radiation exits within the bottom 1/3 of the sidewall in a 52° angle, as seen in Figure 1. This tip has to be renewed after every use.

The study was done as an observational study according to the German regulations (“Medizinproduktegesetz”) which are regulating the medical and ethical conditions for medical treatments. Furthermore, the used laser device and settings have also the approval of the Food and Drug Administration (FDA) for general use in dentistry.

For the laser sessions of the periodontal pockets, the settings for troughing and inner epithelium removal were used, as it is recommended and FDA-approved by the Biolase company:

1.5 Watt, 30 Hz, 11% air, 20% H_2_O, H-mode (pulse duration: 140 *μ*s)

The average power of this setting is 1.2 Watt since the used tip has a calibration factor of 0.8. The pulse energy is 40 mJ and the peak power is 285.71 W.

### 2.4. Microbiological Diagnostic

The pretreatment biological smear was taken from the five deepest pockets in the oral cavity but at least one site in each quadrant as a pool probe. They were gained with sterile paper points that were placed to the deepest part inside the pocket. The paper points were deposited there for five seconds, then taken out, and immediately put in a transport box. All five paper points were transported in the same box.

Carpegen Periodiagnostik in Münster, Germany, did the diagnostics. They use the real time PCR (polymerase chain reaction) to determine the six periodontal pathogens:

- *Aggregatibacter actinomycetemcomitans (A.a.)*
- *Porphyromonas gingivalis (P.g.) *
- *Tannerella forsythia (T.f.) *
- *Treponema denticola (T.d.) *
- *Fusobacterium nucleatum *ssp.* (F.n.)*
- *Prevotella intermedia (P.i.) *

With this special technique, it is possible to determine each germ with a detection limit of 100 cells. This is a very exact quantification so this technique has a very high specificity as well as sensitivity. Those cases, characterized by the number of germs being under the detection limit, were also declared with 100 cells because they are below any physiological effect.

The microbiological analyses lead to conclusions of the

- overall bacterial load in the periodontal pocket,
- the fraction of periodontal pathogens of the bacterial load, which means the counting of the six above-named germs in percentage of the whole bacterial number,
- an accurate counting of each bacterium.

### 2.5. Statistics

The statistical analysis for the pocket depth has been done with descriptive statistics due to the low case numbers. For the microbiological statistics, the significance level was calculated by the Wilcoxon test. *P* values <0.05 were accepted for statistical significance.

## 3. Results

The pocket depth of all sites in each patient was measured before periodontal treatment and six months after treatment. There were 580 measured sites that were treated conventionally and 588 measured sites that were treated with the laser additionally.

The pocket depths before treatment were between 2 and 12 mm. For a better analysis, it is necessary to differentiate this a little bit more. Only patient 9 had pocket depths between 10 and 12 mm. All the other measured sites were in a range of 2–8 mm.

After six months the post-op pocket depths ranged between 1 and 5 mm. All deep sites in patient 9 have been reduced to a depth of four and beneath. Only in patient 5 were there sites that measured 5–7 mm still after treatment. Those deep pockets were only found in the nonlased group.

The mean reduction of the pocket depth was almost the same in the conventionally treated quadrants and the lased quadrants. The mean reduction for the conventionally treated sites was 1.89 mm (standard deviation of 0.76). The mean reduction for the lased sites was 1.92 mm (standard deviation of 0.64). So, there is a slight higher reduction of pocket depth after six months in the lased group (Table 1).

**Figure 2:**
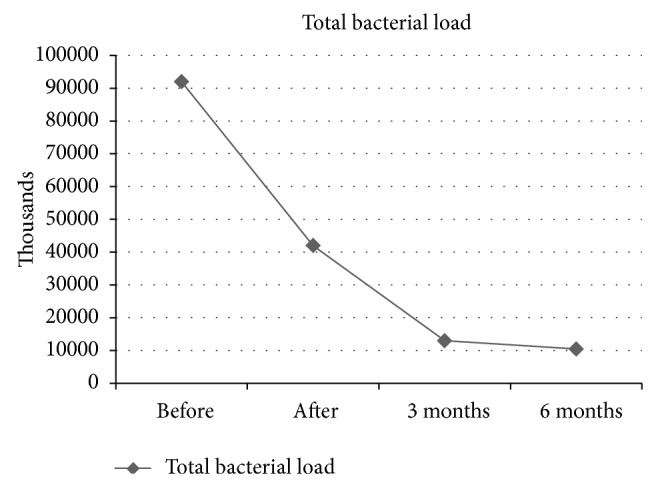


The total bacterial load means all bacteria that were found and counted in the biopsies, not only the six mentioned pathogens. This number, as one can see in Figure 2, was reduced throughout the whole period of examination. Directly after treatment, the bacterial number was reduced from 91.2 · 10^9^ to 42.1 · 10^9^ and, after three months, to 12.9 · 10^9^ 12972500. Six months after treatment, the bacterial load was the lowest value with 10.3 · 10^9^. In Figure 2, the mean values of the bacterial load of all patients are illustrated.

The bacterial quantity was reduced by the laser treatment significantly from the start point to 3 months after treatment (*P* < 0.002) and also to 6 months after treatment (*P* < 0.004).

The percentage decrease of all bacteria in mean for all patients from the disease to 6 months post-op is −88.72%.

*Single Count of Each Pathogen. Prevotella intermedia* was the most present germ in the reviewed oral sites at baseline. Its mean number at baseline was 10.2 · 10^9^. Since it was not present in four patients, the mean number has a very high standard deviation (25 · 10^9^). In patients where the germ was located, it was present in a very high number, up to a maximum of 9.1 · 10^7^. Therefore, this germ was reduced extensively after 3 months and after six months to three orders of magnitude smaller (mean value after six months is 8.7 · 10^5^). The number continues to decrease throughout the whole examination period. Its reduction was significant after 3 months to baseline (*P* = 0.013).

*Porphyromonas gingivalis,* at baseline, was present in all but four patients. After three months, in all other eight patients, the number of* P.g*. was reduced for more than 92%. This is a reduction of two orders of magnitude. After six months, the quantity in two patients increased again. There was still a reduction of more than 99% in three patients; in altogether five patients the number was reduced for more than 85%. The mean number of subsistence of this germ slightly increased from 3 months after treatment to the 6-month post-op examination although there was no significance in the reduction of* P.g*.

*Tannerella forsythia* was reduced in all patients but one extensively directly after treatment from a mean number of 2.4 · 10^6^ to 2.6 · 10^5^. This reduction was even slightly clarified after three months, with a mean number of 1.4 · 10^5^ germs and continuing like so after six months, with 1.3 · 10^5^ in mean. The last microbiological count showed a percentage reduction to baseline of 94.67%. The reduction after three months was significant to baseline (*P* = 0.012) as well as after six months to baseline (*P* = 0.028).

*Treponema denticola* was present in all patients at baseline with a mean number of 3.3 · 10^6^. It was reduced with a high significance from baseline to one day after treatment (*P* = 0.002) and continued to become less at 3 months after treatment with a significance level of *P* = 0.006 to baseline. This continued for the next three months. The reduction after 6 months showed a reduction to baseline with *P* = 0.003. A big reduction was proven again from 2.7 · 10^5^ germs at 3 months to 1.2 · 10^5^ at 6 months after treatment. Its appearance was reduced in all patients. In one patient, this germ was reduced to 100% and in four patients to more than 99% after 6 months.

*Fusobacterium nucleatum* also showed a response to the laser treatment. It was present at baseline in 10 patients with 5.3 · 10^5^ counts in mean. The quantity stayed throughout the whole examination in the same order of magnitude. It was reduced one day after treatment to 2.5 · 10^5^ with a significance level of *P* = 0.001 and was reduced even more after 3 months to 1.3 · 10^5^ (*P* = 0.006). The next examination showed an increased number of 2.4 · 10^7^ which is still in mean −54.64% below baseline but similar to the first post-op day and therefore still a significant reduction to baseline with *P* = 0.003.

The germ* Aggregatibacter actinomycetemcomitans* must be evaluated in a more differentiated way. It was only present in three patients in very unequal numbers. Therefore, it was not reasonable to calculate mean values but to look at them individually.

In two patients, it was reduced in the quantity from baseline. In one patient, its number even increased drastically.

**Figure 3:**
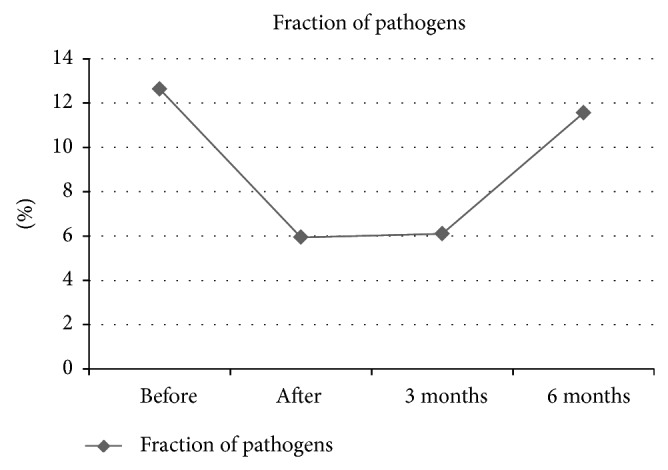


*Fraction of Periodontal Pathogens.* The third part of diagnostics in the microbiological smear is the fraction of the mentioned pathogens from all bacteria inside the periodontal pocket. This is a number in percentage, which has been determined in every smear. The fraction was very diverse for each patient, so there is a high standard deviation (written in brackets) for the mean number. This was especially the case for the 6-month posttreatment result. The mean for all patients was decreased by almost the half from 12, 63% (0.08) to 5, 94% (0.06) directly after treatment and stayed like this for 6, 1% (0.06) 3 months after treatment (Figure 3). Its number grew within the next 3 months then to 11, 56% (0, 11).

## 4. Discussion

Surprisingly, there is no significant difference between both treatment methods considering the pocket depths after six months. Although the antibacterial effect of the laser treatment was very effective and enduring, the pocket depth reduction is similar to the nonlased quadrants. An interesting investigation would be a long-term control after one year or even after three years to determine the long-term role of the microbiological results. This will show whether the lased pockets succumb to less recolonization or are recolonized by only less harmful germs. Crespi et al. [9] found in their 2-year follow-up study a significant difference in the probing depth to favour Er:YAG treatment over conservative treatment with ultrasonic devices.

Schwarz et al. [10] also found a significant better attachment level on those sites that were treated with laser after two years compared with SRP. This might confirm the supposition that laser treatment has a better maintenance effect than all other kinds of conservative periodontal treatment.

Discussing this part of pocket depth reduction, there are two important issues to take into consideration.

Smoking was not an excluding factor for the participating patients. Since this is a key factor in the progression of periodontal disease, whether in proximate studies smokers should be excluded can be discussed. How far nicotine influenced the outcome of this study and inhibited reattachment cannot be considered.

Second issue is the initial pocket depth. Several studies indicate this issue by pointing out the difference in reduction of pocket depth depending on the initial pocket depth [11]. In the present study, initial pocket depths of 2–11 mm were measured and included in the study. P. A. Adriaens and L. M. Adriaens [11] make a difference between “initial pocket depths of 4–6 mm and those over 6 mm.” For a more differentiated conclusion, it would be recommendable to make this parting. So the appropriate therapy could vary whether one has to deal with a chronic periodontitis (Type II) or an aggressive periodontitis (Type III).

The results of this study show a discrete reduction in the bacterial load inside a significant number of examined periodontal pockets as well as a considerable reduction of each examined bacterium itself.

A special focus is on the result of the reduction of the pathogenous bacteria. This is the crucial number for periodontal disease and destroying of periodontal tissue. Since periodontitis is an inflammatory event caused by the examined pathogens, the aim of periodontal treatment is to reduce their fraction inside the periodontal pocket. Nine out of ten examined objects showed a significant reduction of these pathogens, which leads to the assumption that the Er,Cr:YSGG laser has a large impact on the microflora inside a periodontal pocket.

Since we have modern tools, now, like 360° firing tips, the research with those instruments should progress. Maybe different settings or an advanced water-cooling system for this tip could lead to a treatment plan which maybe makes the hand instrumentation secondary or needless. Then, the treatment with laser would be the gold standard for periodontal treatment.

**Figure 4:**
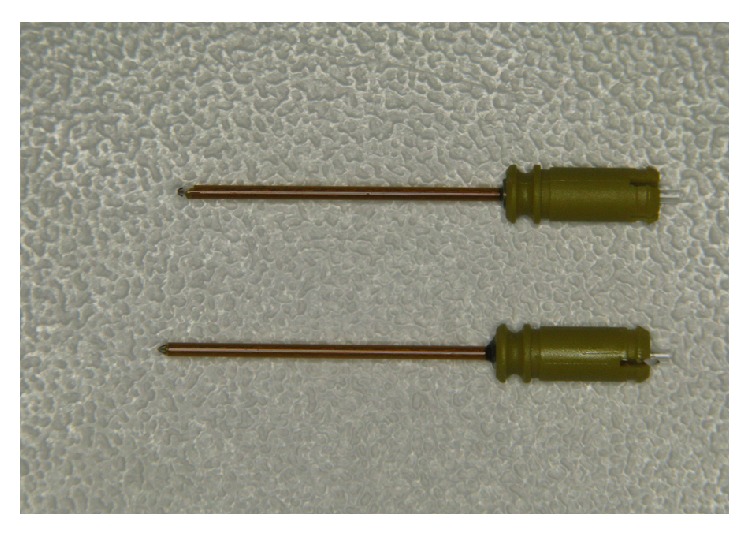


*Application Pros and Cons.* In the past, for pocket curettage, preference had been given to diode lasers and Nd:YAG lasers by clinicians because of their flexible fiber delivery system, which is suitable for all hidden areas inside the oral cavity [12]. But as the operator with this new flexible 14 mm long tip, I must say it is an alleviation to work with this kind of instrument. No danger of breaking the tip inside the pocket appeared during this study and all teeth could have been treated appropriately. It was easy to access all furcations, pocket depths, and distal regions although this tip is for one-way use, since its apex is deformed after treating 4 quadrants. This was evaluated only by visual validation (Figure 4).

During the study, most of the patients felt pain and asked for local anesthesia for the next laser session. Additionally, three of them disapproved the laser treatment for pain that stayed for 1-2 days after every treatment. This pain was described as a dull pinch on the whole gingiva of the laser treated quadrants. In two patients, transformation on the gingival surface appeared. Those white mucosal modifications disappeared after 2 days. They were not hurting or bleeding by contact, although those patients felt this just described pain inside the whole jaw. A reason for this could be the applied energy. Perhaps this was too much energy for periodontal treatment with this tip or at least for the soft tissue side of the periodontal pocket. An explicit advantage of this tip is the decreased amount of straight energy emission, thereby reducing the potential damage to the periodontal ligament when the tip is used vertically [8]. Consequently, a lot of the applied energy penetrates to the surrounding tissue. Another explanation could be the water-cooling. Although water-cooling was used, it can happen that, in deep pockets, the water-cooling does not reach the pocket bottom and laser energy is transformed in too much thermal energy in this region. Since the tip is 14 mm long and sometimes the pocket tissue is very tight, water-cooling may not follow the way into the pocket to the end of the tip.

Further studies for this special Perio tip are necessary to clear this matter.

*Special Role of Actinobacillus actinomycetemcomitans.* There are still a lot of things and functions of* A.a*. that are not known yet, but what is clear is that this organism is capable of causing marked alterations in its host as a result of its powerful toxins and its ability to adhere to host cells and to enter into them and travel through them [13]. Laser due to the thermal energy that is transferred to the tissue kills this germ. This may be an accusation for human treatment. But considering that the heat that is needed for deleting* A.a*. is about 42°C, the laser energy that is needed is below any concerning level.

In the present study, only in four patients was* A.a*. present at baseline. In three cases, its quantity was reduced at six months after treatment. In one patient, it could be reduced under the detection limit. This is a very good result compared to other studies that have examined periodontal treatment with or without laser, but it demands more evidence based examinations since now in research only therapies using amoxicillin and metronidazole in combination with full-mouth scaling and root planning were able to reduce* A.a*. in all cases [12, 14].

*Abuse of Antibiotics.* A lot of diverse studies of conservative periodontal treatment have shown that mechanical treatment itself, even when performed with sonic or ultrasonic devices, in some, especially severe, cases of periodontal disease, does not lead to a complete healing or a satisfactory reattachment. This is due to the fact that mechanical treatment does not delete the periopathogenes adequately. Herrera et al. [15] reviewed the beneficial effect of adjunctive systemic antibiotics on the clinical outcomes of nonsurgical periodontal treatment. The gold standard for this adjunctive medication is a treatment with amoxicillin and metronidazole as a combination dose for seven days. van Winkelhoff and Winkel state in 2009 [16] that this mentioned antibiotic combination still seems to have the best clinical outcome.

However, a big issue in the application of systemic antibiotics is a multiplicity of possible side effects. Even mild secondary effects can lead to the patient not taking the medication properly as prescribed, thereby decreasing the efficacy of the medication [17].

Another problem in medication with systemic antibiotics is that many bacteria become more and more resistant to antibiotics or find ways to guard themselves against these substrates, especially those germs that are organized in a biofilm [18]. van Winkelhoff and Winkel [16] are convinced of the necessity of taking a bacterial profile before each periodontal treatment, since they found that treatment of* P. gingivalis*-negative patients with antibiotics may be considered an overtreatment. They say that the subgingival microbial profile at baseline may be one determining factor of the clinical effects of systemic antimicrobial therapy. Facing the progress of resistance of bacteria against antibiotics, it is an essential tool in periodontal treatment to take a bacterial profile before treatment and to decide in each case whether there is not an equal or even better treatment plan for each individual case. This study shows a new effective way of treating chronic periodontitis without antibiotics, excepting germ pools that contain* A.a*.

Like Herrera et al. [15], I would like to still stick to the old postulate that, due to the problems related to their indiscriminate use like systemic side effects and increase in bacterial resistance, the use of systemic antimicrobials in periodontitis should be restricted to certain patients and certain periodontal conditions. Moreover, when antibiotics are prescribed, they should be given within the context of biofilm disruption and related to the properties of the target.

If laser treatment helps to avoid loading human systems with antibiotics only in some cases, it is already a very good step for the health of the patient.

## 5. Conclusion

With the laser, it is possible to deepithelise the flap, respectively, the inner-epithelium lining, in order to increase the distance for the epithelial cells to travel and to allow the periodontal ligament cells to reach the radicular surface first. This will avoid a so-called long epithelial attachment.

Not only the acceptance of laser supported periodontal treatment by the patients, which helps to encourage the compliance, but also the very high bactericidal effect as it is shown in this and in other studies, makes the laser treatment an indispensable part of periodontal treatment. Furthermore, as it seems to be in the present study and as it has been reported that the bactericidal effect of laser treatment has a better maintenance effect and therefore a better wound healing effect than SRP alone [19]; the laser assisted treatment may be a definitely better treatment as the nonsurgical periodontal treatment without laser. The clinical and microbiological improvements may be a combination of a beneficial conditioning of the root surface, mechanical disorganization of the biofilm, and reduction in viable bacteria as well as inactivating bacterial toxins [20]. Therefore, it can be assumed that the repetition laser sessions may be done with less energy, since scaling and root planning work on the hard tissue side is done and only the antimicrobial part is of importance.

The new RFPT 5-14 tip is characterized by a good handling for the operator, a low fracture risk, and, due to its flexibility, a good access to furcations, distal regions, and other difficult parts inside the oral cavity. However, more studies have to follow in order to specify the laser settings, particularly the water-cooling, to reduce the patients' pain and the soft tissue reaction.

Those side reactions should be remedied in order to find a new omnipotential tool for periodontal treatment, since the 360° firing tip offers the opportunity to efficiently treat the soft tissue site of the periodontal pocket and at the same time clean and decontaminate the hard tissue side of the tooth. This is a clear advantage of this new tip in connection with the wavelength of 2,780 nm.

## 6. Summary

The results in this study prove that the Er,Cr:YSGG laser with a 360° firing tip is able to reduce pathogenous microorganisms in the periodontal niche significantly. Microbiological examinations showed a strong reduction of the whole bacterial amount in the pocket as well as the number of each periodontal pathogen. This result stayed true until 6 months after treatment. The pocket depth of the treated sites showed a better reduction after using the laser compared with the nonlased sites.

## Figures and Tables

**Table 1:** Clinical Parameters at baseline and 6 months after treatment.

	Baseline	6 months	Mean reduction
Test group	5.28 ± 0.91	3.36 ± 0.72^a^	1.92 ± 0.64
Control group	5.11 ± 0.83	3.22 ± 0.52^a^	1.89 ± 0.76

^a^ *P* > 0.05; *P* values represent statistically significant changes from baseline within each group.
